# Gene Expression Analysis in *gla*-Mutant Zebrafish Reveals Enhanced Ca^2+^ Signaling Similar to Fabry Disease

**DOI:** 10.3390/ijms24010358

**Published:** 2022-12-26

**Authors:** Hassan Osman Alhassan Elsaid, Håkon Tjeldnes, Mariell Rivedal, Camille Serre, Øystein Eikrem, Einar Svarstad, Camilla Tøndel, Hans-Peter Marti, Jessica Furriol, Janka Babickova

**Affiliations:** 1 Department of Clinical Medicine, University of Bergen, 5021 Bergen, Norway; 2 Department of Medicine, Haukeland University Hospital, 5021 Bergen, Norway; 3 Computational Biology Unit, Department of Informatics, University of Bergen, 5021 Bergen, Norway; 4 Department of Pediatrics, Haukeland University Hospital, 5021 Bergen, Norway; 5 Institute of Molecular Biomedicine, Faculty of Medicine, Comenius University, 811 08 Bratislava, Slovakia

**Keywords:** *gla*, alpha-galactosidase A, zebrafish, cardiac involvement, Fabry disease, calcium signaling, oxidative stress

## Abstract

Fabry disease (FD) is an X-linked inborn metabolic disorder due to partial or complete lysosomal α-galactosidase A deficiency. FD is characterized by progressive renal insufficiency and cardio- and cerebrovascular involvement. Restricted access on Gb3-independent tissue injury experimental models has limited the understanding of FD pathophysiology and delayed the development of new therapies. Accumulating glycosphingolipids, mainly Gb3 and lysoGb3, are Fabry specific markers used in clinical follow up. However, recent studies suggest there is a need for additional markers to monitor FD clinical course or response to treatment. We used a *gla*-knockout zebrafish (ZF) to investigate alternative biomarkers in Gb3-free-conditions. RNA sequencing was used to identify transcriptomic signatures in kidney tissues discriminating *gla*-mutant (M) from wild type (WT) ZF. Gene Ontology (GO) and KEGG pathways analysis showed upregulation of immune system activation and downregulation of oxidative phosphorylation pathways in kidneys from M ZF. In addition, upregulation of the Ca^2+^ signaling pathway was also detectable in M ZF kidneys. Importantly, disruption of mitochondrial and lysosome-related pathways observed in M ZF was validated by immunohistochemistry. Thus, this ZF model expands the pathophysiological understanding of FD, the Gb3-independent effects of *gla* mutations could be used to explore new therapeutic targets for FD.

## 1. Introduction

Fabry disease (FD) is a rare lysosomal storage disorder affecting multiple organs. Organ dysfunction often correlated with accumulation of globotriaosylceramide (Gb3) in lysosomes in different cells [1]. Clinical symptoms are common in both genders and may appear in early childhood [2]. FD is classified into classical and non-classical phenotypes with a different degree of deficiency of α-GAL, the lysosomal enzyme responsible for Gb3 degradation [3]. While the classical form of the disease is characterized by null α-GAL activity, the non-classical phenotype is characterized by residual α-GAL activity and the absence of classical FD symptoms like acroparesthesia and low sweating ability. Cardiac and renal dysfunction is common in adult FD, with impaired quality of life and premature death [4,5].

In early FD stages, renal involvement is clinically asymptomatic, commonly monitored by measuring albuminuria/proteinuria and glomerular filtration rate (GFR) [6]. However, these tests have a low sensitivity for detecting early kidney damage, and aberrant readings frequently only represent late indicators of renal disease associated with irreparable structural damage [7] not benefiting from FD specific treatment like enzyme replacement therapy (ERT) [8]. Thus, they have limited sensitivity for early detection and monitoring of FD nephropathy, essential to preserving renal function [9,10].

Accumulation of Gb3 and its deacylated form lysoGb3 is involved in various FD pathophysiological manifestations [11,12,13]. However, slow, progressive tissue deposition is insufficient to explain the gradual onset of organ dysfunction or adverse outcome [14,15,16]. For instance, plasma lysoGb3 levels fail to conclusively identify patients with milder phenotypes, particularly in females [17]. Other factors beyond Gb3 accumulation and lyoGb3 exposure may influence FD pathogenicity [18,19]. We have recently shown that renal damage is evident in a Gb3-independent FD zebrafish model [20].

Early biomarkers of FD nephropathy have been investigated in adult patients, with promising results. However, most studies were conducted by using proteomic or metabolomic technology either in urine or in plasma [21], whereas RNA sequencing in tissue has more rarely been performed [8].

More importantly, no studies have been conducted to explore potential mRNA FD biomarkers associated with α-GAL deficiency in Gb3-free conditions. Such investigations might pave the way towards earlier detection of the disease prior to the development of the wide range of organ damage caused by Gb3 accumulation. It is worth mentioning that in addition to the injuries caused by the Gb3 accumulation in FD, other injuries or reactions resulting from the mutant protein can also be attributed to the disease cause, for example the unfolded protein response (UPR) [22]. Recently, it has been shown that such response can cause endoplasmic reticulum stress, mitochondrial stress and lysosomal stress before the Gb3 accumulation effect [23,24].

We have previously shown the similarity of zebrafish’s *gla* and human α-Gal [20]. Therefore, we used a Gb3 synthase-free-*gla-* mutant zebrafish (ZF) as a FD model to investigate the Gb3-independent gene expression signature.

## 2. Results

### 2.1. RNA Sequencing

**Figure 1:**
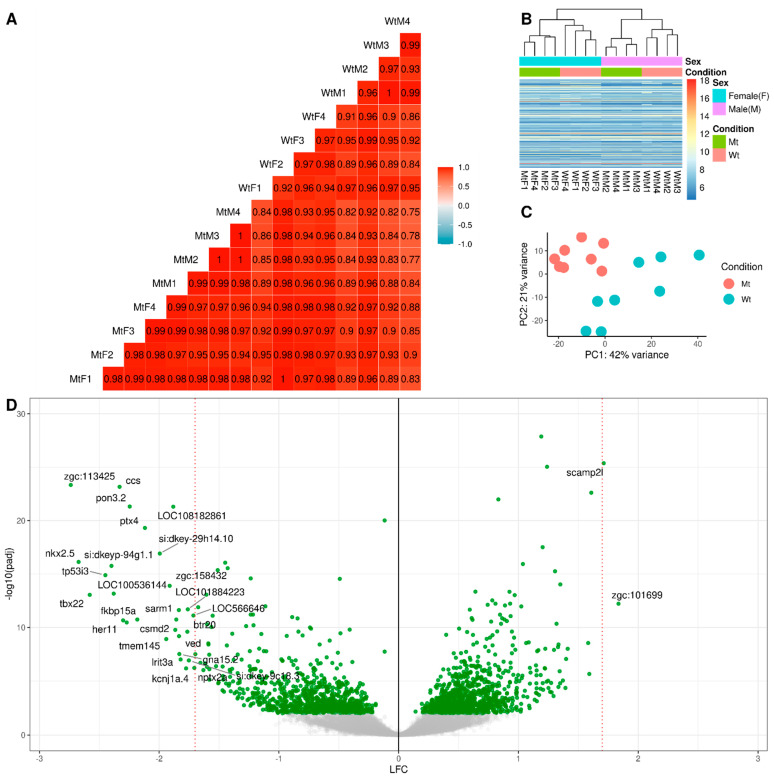
Quality Control and Fold Change. Total samples (*n =* 16), with sex-matched groups: MtM: Mutant Male (*n =* 4), MtF: Mutant Female (*n =* 4), WtM: Wild type Male (*n =* 4), WtF: Wild type Female (*n =* 4). (**A**) Pearson Correlation for pairwise comparison of mRNA FPKM between samples, rounded to 2 decimals; (**B**) Heatmap clustering of variance stabilized counts for all genes binned into five hundred clusters, showing that both sex and condition are clustering together; (**C**) PCA analysis of variance stabilized counts for all genes, with batch correction for both Sex and Replicates; (**D**) Volcano plot of Log2 fold change (LFC) for all genes using DESeq2 differential expression analysis, with resulting −log10 adjusted *p*-values. (Negative LFC means LFC (Mutant Mt) < LFC (Wild type Wt). A high confidence set of genes are displayed with gene symbols (adjusted *p*-value < 0.000001 and absolute value of LFC > 1.7).

First, we performed a quality control of the RNA-seq data obtained from zebrafish (ZF) kidney samples (*n* = 16). Besides expected sex-related differences, this analysis showed a very high (r^2^ > 0.91) correlation among mutant (M, *n =* 8) and wild type (W, *n =* 8) samples, consistent with high homogeneity and data reproducibility (Figure 1A). Most importantly, M and WT samples were indeed clustering better together than between each other, as shown by variance stabilized counts using both a clustering heatmap and a 2-dimensional principal component analysis (PCA) (Figure 1B,C), thereby suggesting that the expression of defined gene subset(s) clearly separated sample groups.

### 2.2. Differential Gene Expression Analysis

Gene expression changes in ZF kidney tissues were then comparatively analyzed in detail. In total, 22,646 genes were successfully identified, and 4042 of them showing high FDR confidence (padj < 0.05) were used for further analysis. A total of 2224 genes were differentially expressed (FDR ≤ 0.05, FC ≥ 0.5 and ≤−0.5) in mutant (M) compared to wildtype (WT) ZF with 1209 downregulated, and 1015 upregulated in MT vs. WT. Figure 1D reports the global differential expression pattern, showing that a higher number of downregulated genes displayed very high Log2 Fold Change.

**Figure 2:**
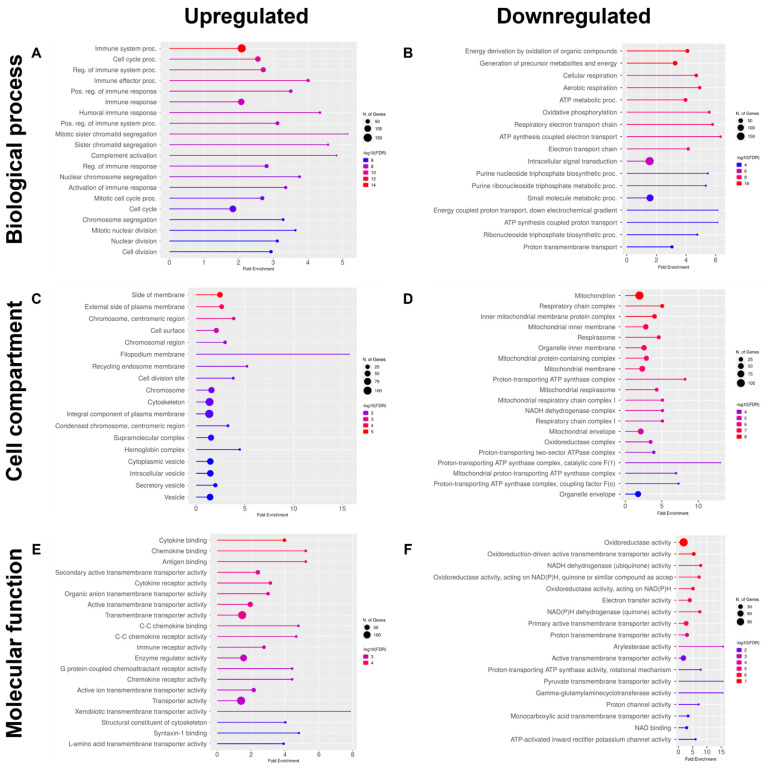
Gene ontology (GO) enrichment analysis of pathways upregulated and downregulated in renal tissues from the mutant, compared to wildtype ZF. Data refers to GO Biological Process (BP: (**A**) and (**B**), respectively), Cellular component (CC, (**C**) and (**D**), respectively) and Molecular function (MF: (**E**) and (**F**), respectively). In all cases, the twenty most enriched pathways are reported. FDR ≤ 0.05.

Differentially expressed genes (DEGs) in M vs. WT ZF renal tissues were first analyzed by Gene Ontology (GO) term enrichment. A high number of genes markedly upregulated in M compared to WT samples were involved in different phases of the immune response, including activation, regulation and effector functions, as indicated by GO terms of biological process (BP) analysis (Figure 2A). On the other hand, downregulated genes were relevant to energy production and consumption, aerobic respiration and oxidative phosphorylation (Figure 2B).

In cell compartment (CC) terms, genes upregulated in M vs. WT specimens mostly encoded proteins expressed on the external sides of cell membranes, in the cytoskeleton and cytoplasmic vesicles (Figure 2C). In contrast, downregulated genes mainly encoded proteins associated with the mitochondrial compartment, including mitochondrial inner membranes and involved in the respiratory chain (Figure 2D).

Finally, molecular function (MF) term analysis showed that genes upregulated in M samples encoded proteins widely involved in cytokine and chemokine binding and signaling, and antigen binding, in addition to different transporter activities (Figure 2E). On the other hand, downregulated genes mainly encoded proteins involved in oxidoreductase activity, electron transfer activity, NAD(P)H dehydrogenase activity, and pyruvate transmembrane transporter activity (Figure 2F).

**Figure 3:**
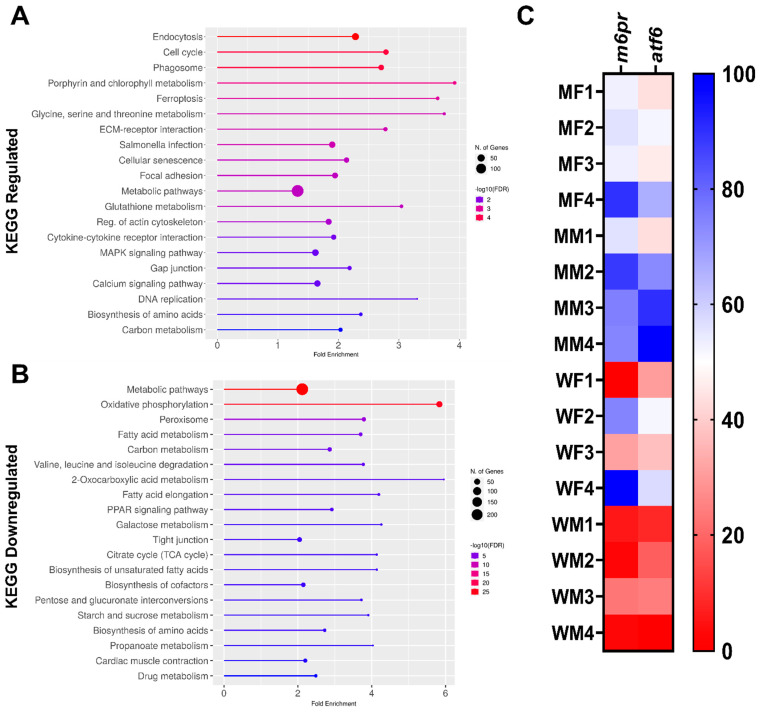
KEGG pathway enrichment analysis and heatmap of m6pr and atf6 genes in mutant compared to wildtype ZF. (**A**) KEGG pathways associated with the upregulated genes; (**B**) KEGG pathways associated with the downregulated genes; (**C**) heatmap (percentage column normalized, where dark blue is the maximum per column) showing the expression of the selected downregulated genes m6pr and atf6.

KEGG analysis revealed dysregulated genes associated with various pathways in M, compared to WT kidney samples. Upregulated genes were relevant to endocytosis, cell cycle, phagosome functions, ferroptosis, ECM-receptor interaction, cellular senescence, focal adhesion, glutathione metabolism, and calcium signaling pathways (Figure 3A). In contrast, downregulated genes were mainly involved in oxidative phosphorylation, in defined metabolic pathways, including carbon and fatty acid metabolism, and peroxisome functions (Figure 3B).

In association with *gla* mutation leading to distorted α-Gal activity, we also investigated the mannose-6-phosphate receptor (m6pr) which transports the enzyme to the lysosome and the activating transcription factor 6 (atf6) which is involved in the UPR cellular protein homeostasis. Our gene expression analysis revealed upregulation in both m6pr and atf6 (Figure 3C).

### 2.3. Validation by Immunohistochemistry and Western Blot

**Figure 4:**
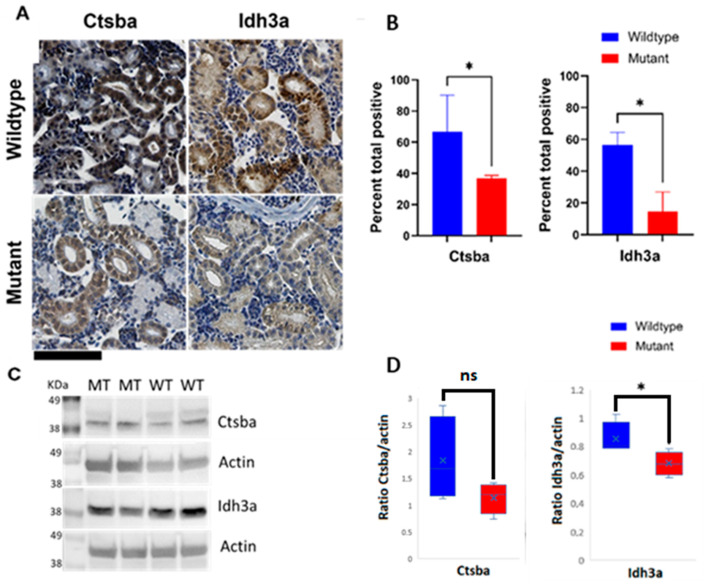
Immunohistochemical and Western blot detection of selected proteins in kidneys from WT and M ZF reveals protein expression disturbances in mitochondria and lysosomes. (**A**) Representative IHC staining specific for mitochondrial marker isocitrate dehydrogenase (NAD^(+)^)3 alpha (Idh3a) and lysosomal marker cathepsin B (Ctsb) in kidney tissue sections from WT and M ZF (**upper right** and **upper left** panels, and **lower right** and **lower left** panels, respectively; (**B**) Quantification of immunohistochemical staining of sections from WT and M ZF kidneys. Signal intensity is significantly higher in WT than in M for both proteins. (**C**) Representative immunoblots of Ctsb and Idh3a in kidney tissue section from WT and M ZF; (**D**) Quantification of immunoblots from WT and M ZF kidneys. (Mann–Whitney test U * *p* < 0.05). Wildtype (WT), mutant (M). ns: not significant. Scale bar (**bottom left** corner, in black) = 100 µm.

To validate gene expression data, we performed immunohistochemistry (IHC) and Western blot (WB) of proteins encoded by genes differentially expressed in renal tissues from M and WT ZF. The selection of proteins was based on the extent of the dysregulation of encoding genes and the availability of commercially validated ZF antibodies. Two proteins, isocitrate dehydrogenase subunit alpha (Idh3a), expressed in mitochondria, and cathepsin B (Ctsb), expressed in lysosomes, met these criteria (Figure 4A). A semiquantitative immunohistochemical and band intensity analysis demonstrated reduced average signals in kidneys from M compared to their WT counterparts (Figure 4B,C)), consistent with expression patterns of the corresponding genes.

## 3. Discussion

In our previous study, we developed an innovative model of FD in ZF [20], amenable to investigations addressing pathogenetic mechanisms and identifying potential drug targets. Additionally, we have shown that elevated oxidative stress, disrupted glutathione metabolism, dysregulated autophagy and differences in plasma metabolites and distorted mitochondrial morphology in our model (article under review). Here, we further expand the pathophysiological understanding of FD and show that a thorough analysis of gene expression in kidneys from *gla*-mutant and WT ZF reveals specific patterns of potential clinical relevance.

Gene ontology (GO) data show that genes associated with different aspects of the immune response are highly significantly upregulated in M ZF. This data is supported by the analysis of molecular function (MF), showing upregulation of genes associated with chemokine and cytokine activity, and response to antigens.

Similarly to other lysosomal storage disorders, previous studies have repeatedly suggested an important role of immune response in FD pathogenesis [25,26]. However, immune system activation was generally attributed to Gb3 accumulation [27,28], leading to invariant natural killer T cells (iNKT) activation. In this respect, our data provide important new information. Indeed, while both innate and adaptive immune systems are functional in ZF [29,30], mutant *gla* knock-out ZF does not produce Gb3 [20]. Therefore, our results indicate that immune system activation closely resembling that observed in FD patients [31,32] takes actually place in the absence of Gb3 as well, and set the stage for the identification of novel “immunostimulatory” compounds [33] of potential clinical relevance in FD. Accordingly, *zgc:101699* gene, the second most upregulated gene in kidneys from M ZF, encodes a phospholipase, belonging to a protein family highly overexpressed upon inflammation [34,35].

Our group’s transcriptomics analysis from kidney biopsy revealed differences between FD patients at three time points (baseline, five years post-ERT, and 8–10 years post ERT) and healthy controls [36,37,38]. Gene Set Enrichment Analysis in the glomerular compartment demonstrated enriched gene sets of extracellular matrix, EMT, fibrosis, and immune response. The early ERT intervention seemed to return the upregulated pathways to normal, similar to the control samples [36]. However, these enriched pathways remained high in the long-term, e.g., 10 years of ERT [37,38]. Our results are in line with these investigations. Furthermore, we have also identified that complement component 1, q subcomponent, C chain gene (*c1qc*) is significantly upregulated in the mutant fish compared to the wild type, which has already been shown by Strauss et al., [37] and Heo et al., [39]. While Eikrem et al. and Strauss et al. have shown these results in humans in the presence of Gb3, we have shown that similar results can also be achieved in its absence. Our results indicate that other pathways might be responsible for triggering and maintaining such pathways independently of Gb3, which is supported by previous research [40].

Interestingly, we have observed upregulation of *m6pr* at the gene expression level in the mutant fish kidney compared to the wildtype, contrary to the recent finding of Frustaci et al. in endomyocardial biopsy [41]. M6PR is the lysosomal receptor for α-GAL [42,43]; therefore, its downregulation can negatively impact the efficacy of the ERT. The contrasted regulation can be possibly attributed to the post-translation protein modification. Our results give the first wet lab evidence on the previously suggested Gb3 independent effects and strongly suggest that Gb3 independent effects should be extensively investigated.

Our results show upregulation of *atf6* gene consistent with previous findings in FD [24]. The UPR restore cellular protein homeostasis by activating three distinct sensors: double-stranded RNA-activated protein kinase (PKR)–like ER kinase (PERK), inositol requiring enzyme (1IRE1), and activating transcription factor 6 (ATF6) [44]. These in turn activate downstream pathways targeted at decreasing protein synthesis and enhancing ER-associated folding and destruction [44,45]. Chronic stimulation of the UPR, as in the case of continuous synthesis of a mutant protein, can result in cell death and/or inflammatory activation, and it has been linked to various conformational disorders including lysosomal storage diseases [46,47].

Most notably, GO analysis of genes downregulated in M ZF indicates that, among others, genes involved in Cellular respiration, Aerobic respiration and Oxidative phosphorylation are markedly affected. This data suggest a prevailingly anaerobic, glycolytic metabolism, representing an inefficient, emergency energy production pathway, in M ZF kidneys, consistent with an ongoing immune system activation [48].

KEGG analysis further supports these findings, since pathways associated with inflammation, such as phagosome activation, endocytosis and ferroptosis are upregulated in kidneys from M ZF. Possibly as a consequence of immune stimulation, calcium signaling pathway is also upregulated in M ZF as calcium is well known key regulator in the immune cells [49,50]. Remarkably, previous studies using *GLA* mutant human inducible pluripotent stem cells (iPSC) in a kidney organoid, have shown an increased calcium influx into the cytoplasm in response to oxidative stress [51]. Additionally, in Fabry knockout murine tissues, expression of S100 calcium-binding proteins A8 and A9 (also known as MRP8 and MRP14), was markedly elevated at the gene and protein levels [52]. Importantly, S100A8/A9, Ca^2+^ sensors involved in cytoskeleton remodeling and arachidonic acid metabolism, are expressed constitutively by neutrophils and monocytes and are actively produced during inflammation, promoting leukocyte recruitment and cytokine production [53].

In line with GO data, KEGG analysis confirms that metabolic pathways, and, in particular oxidative phosphorylation, are downregulated in kidneys from M ZF. In addition, the expression of a variety of cytochrome genes, typically detectable in mitochondria, but collectively included in the cardiac muscle contraction pathway in the KEGG analysis, is also markedly downregulated in kidneys from M ZF. Taken together these observations delineate a mitochondrial stress scenario, possibly associated with Ca^2+^ signaling dysregulation, in line with our previous findings, and with previous reports in FD [51,54,55], and in other heart and muscle disorders [56].

Based on transcriptome data and on the availability of ZF-specific reagents, we tested the expression of selected proteins in kidneys from M and WT ZF to validate our findings at the protein level.

Isocitrate dehydrogenase catalytic subunit alpha (Idh3a) is a mitochondrial protein encoded by a gene downregulated in kidneys from M compared to WT ZF. Idh3a promotes ATP production by catalyzing oxidative decarboxylation of isocitrate to 2-oxoglutarate. Downregulation of this enzyme is also known to affect neurotransmission [57]. Consistent with gene expression data, Idh3a protein expression was also decreased in M, as compared to WT ZF kidney sections, as shown by both immunohistochemistry and Western blot.

The lysosomal/cytoplasmic vessel cysteine protease cathepsin Ba (*ctsba*), an orthologous to human *CTSB* gene was also downregulated at both the gene and protein level. CTSB is a key player in lysosomal homeostasis and its dysregulation has been linked to variable lysosomal abnormalities [58,59,60]. CTSB downregulation results in autophagosome accumulation due to compromised lysosomal clearance [61]. In general terms, dysregulation of cathepsins (CTSs) expression and/or activity, impairing cellular homeostasis, leads to a variety of human diseases, including, cardiovascular diseases, neurodegenerative disorders, and kidney dysfunctions [58]. However, CTSB expression has not been associated with FD so far. In Niemann-Pick type C (NPC) disorder, a rare neurodegenerative disorder, inhibition of this protease results in lysosomal dysfunction [62]. CTSB resides in the lysosome, the cytosol and the extracellular space, where it participates in many functions, e.g., inflammasome triggering, apoptosis, and extracellular matrix degradation [60]. The CTSB has been proposed as a potential biomarker as it can be measured in urine and plasma [63,64], and previous studies have shown its downregulation is associated with the renal tubular injury [64,65,66,67,68], even at an early stage in life [63].

Contrary to our findings, other studies revealed elevated lysosome-associated protein 1 and 2 in FD patients [69,70]. A possible explanation to this contradiction is that in these studies, lysosomal dysregulation is attributed to the Gb3 accumulation while in our study we highlight the Gb3-independent effect in a complete Gb3-free environment. We therefore suggest that the effect seen in our study is triggered through alternative pathways, for example the UPR which has recently been reported in FD [23,24].

Limitations of our study should be acknowledged. In particular, in ZF, kidney is also a major hematopoietic organ [71,72,73,74], and, as such, it physiologically includes myeloid cells at different maturation stages. However, genes encoding cytokines and chemokines are highly upregulated in kidneys from M, as compared to WT ZF, thereby ruling out the possibility that the mere nature of the experimental model accounts for our results. Moreover, admittedly, our study exclusively focuses on gene expression in ZF kidneys, whereas other organs are also affected in FD. However, alterations of kidney functions do represent some of the most common FD symptoms. On the other hand, the scarce availability of ZF-specific antibodies has limited the validation at the protein level.

Nonetheless, our findings show that even in the absence of Gb3 and lysoGb3, specific alterations of gene and protein expression may be detected in M ZF. While validating this model as an important tool to explore FD pathogenesis and to identify new drug targets, our data pave the way for studies investigating novel, clinically relevant FD markers.

In conclusion, for the first time we have demonstrated a Gb3-free impact similar to FD in humans using our gla-mutant zebrafish. Independent of Gb3, calcium ion flux disruption and altered mitochondrial and lysosomal pathways can be established and maintained. Our findings therefore support the hypothesis mechanisms beyond Gb3 accumuation are involved in the onset and maintenance of these processes in FD.

## 4. Materials and Methods

### 4.1. Ethical Approval

FOTS ID 15256 was granted by the Norwegian Food Safety Authority (Mattilsynet) for this study. All procedures were performed following the Zebrafish Facility protocol at the University of Bergen (UiB), by using the AB/Tübingen (AB/TU) strain of ZF and the *gla*-mutant ZF previously generated by our group [20].

### 4.2. Zebrafish Maintenance and Sample Collection

Eggs, embryos, larvae, juveniles, and adult fish were handled in compliance with applicable national and international standards, according to ZF facility regulation at the University of Bergen. Under normal laboratory conditions, an adult (90+ days post-fertilization dpf) wild-type ZF was held at 28 °C on a 14 h light/10 h dark period. Standard spawning protocol (www.zfin.org, accessed on 18 October 2022) was followed by egg harvesting. Eggs were stored in an E3 medium containing 0.01% methylene blue after harvesting. Embryos and larvae were incubated at 28 °C until 5 dpf. Current regulation does not require permission for testing on ZF embryos before the free-feeding stage (5 dpf). Instead, according to the ZF facility rules, all invasive pain-causing interventions on stages older than 5 dpf was performed under anesthetic conditions. For sample collection, adult ZF were humanely euthanized in 300 mg/L tricaine methanesulfonate MS222 Sigma-Aldrich A-5040 (Steinheim, Germany), and then dissected open. Kidneys were exposed after discarding the viscera under cold 1X PBS Life Technologies AM9625 (Waltham, MA, USA), removed and placed into clean RNAse-free tube prefilled with RNAlater™ Stabilization Solution AM7021(Waltham, MA, USA). Three kidneys were pooled per sample. Samples were kept overnight at 4 °C and stored at −80 °C until further RNA extraction take place.

### 4.3. RNA Extraction and Sequencing

Total RNA was extracted from whole kidneys (N *=* 8/group) using RNeasy Mini Kit Cat. No. 74104 (Qiagen, Hilden, Germany) following the manufacturer’s protocol. Quantification of RNA concentration and purity was determined using NanoDrop One/One ND-ONE-W (Thermo Fisher, Waltham, MA, USA) and Qubit 3 Q33216 (Thermo Fisher, Waltham, MA, USA). Concentration with nanodrop and qubit with a correlation of 0.99 and purity with nanodrop with ratios 260/280 2.08 ± 0.02 and 260/230 1.21 ± 0.66. The integrity of the RNA, cDNA library construction and Illumina sequencing were performed by NovoGene (Beijing, China) as described previously [75].

### 4.4. RNA Sequences Analysis

Raw data (FASTQ format) were processed using the STAR aligner and resulting files were processed with R/Bioconductor packages (R version 4.2). After initial QC checks in fastp, read mapping was done with STAR (version 2.7.4a). STAR used the indexed version of Danio rerio: GRCz11 Ensembl patch 101. The final analysis was performed using the RNA-seq pipeline in ORFik [76,77,78,79]. The primary Ensembl isoform per gene was used (usually the isoform with the longest coding sequence CDS). Number of reads per gene for each sample were counted using GenomicRanges (version: 1.48.0) count Overlaps (a hit is >1 nucleotide of read overlapping any gene exon on the same strand) [80]. In the pairwise comparison (M vs. WT), DEGs were identified by using the DESeq2 R package (version 1.36.0) and selected using a Wald’s test with filter criterion of FDR < 0.05 and FC > 0.5 [81]. FC values were shrunk using a Normal prior, with the remaining options for DESeq2 set to default values. Experimental design was set to: ~condition (wt vs. mt) + sex (M vs. F) and contrast as condition, to only acquire differential expression from treatment. Variance stabilized counts for PCA and heatmap analysis in Figure 1 were done with the vst() function from DESeq2, using the default Gamma GLM fit for the mean/dispersion relationship. Visualizations from R were done using the packages ggplot2 [82]. Processing script can be found at: https://github.com/Roleren/fabry_article_code, (accessed on 18 October 2022). RNA-seq data are available at ENA project id number: PRJEB55250.

Gene ontology (GO) and KEGG pathway analyses were performed using ShinyGO 0.76 [83]. Genes/pathways with adjusted *p*-value < 0.05 were considered as significantly differentially enriched. Heatmap for normalized count (FPKM: RNA-seq Fragments overlapping gene per Million fragments in library per Kilobase of exon in gene) was produced using GraphPad Prism V 9.2.0. We used FC of ±0.8 as produced FC using DESeq2 more realistic [84,85] as it focuses on analysis strength, not the expression [81].

### 4.5. Immunohistochemistry

Kidney samples of adult ZF (90+dpf) were used. N = 12 (6/genotype, 3 males, 3 females). IHC was performed as previously described [86] with slight modifications for each antibody. Heat-induced antigen retrieval was performed for 3 min in Dako Target Retrieval Solution, pH 6 (CTSB) and pH 9 (IDH3a) (Glostrup, Denmark). Incubation with primary antibody was performed for one hour at room temperature. Antibodies used were IDH3a GTX124431 (1:200) from GeneTex (Irvine, CA, USA) and CTSB M1506-1 (1:1000) from HUABIO (New Boston, MI, USA). For negative controls, the primary antibody was omitted. Slides were scanned with ScanScope XT^®^ Aperio (Vista, CA, USA) at ×40 resulting in a resolution of 0.25 micrometer per Pixel. Digital slides were viewed in ImageScope 12 (Waltham, MA, USA).

IHC positivity for each antibody was quantified using the color deconvolution algorithm version 9.1 (Vista, CA, USA) after adjusting the default parameters to DAB staining. Total percentage of positive pixels was used as a visualization parameter and statistics was performed using GraphPad Prism V 9.2.0 (San Diego, CA, USA). Values are presented as mean ± SD and Mann–Whitney test was used to assess statistical significance. Differences were considered significant with *p*-values < 0.05.

### 4.6. Western Blot

Kidney samples of adult ZF (90+dpf) were used. N = 12 (6/genotype, 3 males, 3 fe-males: pool of three kidneys/sample). Protein extraction was performed using RIPA buffer catalog no. R0278 (Sigma-Aldrich, Steinheim, Germany) with the addition of complete protease inhibitor 4693116001 (Roche, Basel, Switzerland) and phosphatase inhibitor cocktail P5726 (Sigma-Aldrich, Steinheim, Germany)). Protein concentration was determined using Pierce BCA Protein Assay Kit (Thermo Scientific, Waltham, MA, USA). Proteins were separated in Bolt 4–12% Bis-Tris Plus electrophoresis gels and transferred to nitrocellulose membranes using iBlot 2 System (Invitrogen, Waltham, MA, USA). Membranes were blocked with 5% BSA in 1X PBS containing 0.1% Tween-20 and then incubated overnight with rabbit polyclonal Anti-Idh3 antibody GTX124431 (GeneTex, Irvine, CA, USA) at 1:1000 dilution and mouse-monoclonal Anti-Ctsb antibody M1506-1(HUABIO,New Boston, MI, USA ) at a dilution of 1:5000. Anti-beta-actin A3854 (Merk,Rahway, NJ, USA) was used as loading control proteinat a dilution of 1:5000.

SeeBlue Plus2 Pre-stained Protein Standard LC5925 (Invitrogen, Waltham, MA, USA)) was used to visualize protein molecular weight. The blots were washed three times with a wash buffer (PBS, 0.1% Tween-20) and then incubated for 1 h either with goat anti-rabbit ab205718 or goat anti-mouse secondary ab205719 HRP-linked antibodies (Abcam, Cambridge, UK). The blots were washed again and developed using Pierce ECL Plus Western blotting substrate (Thermo Fisher, Waltham, MA, USA). Chemo-luminescence signals were assessed using ChemiDoc Imaging System (Bio-rad, Hercules, CA, USA).

## Data Availability

RNA-seq raw data project id: PRJEB55250; Data processing scripts: https://github.com/Roleren/fabry_article_code, accessed on 18 October 2022.
